# Medication adherence and survival among hospitalized heart failure patients in a tertiary hospital in Tanzania: a prospective cohort study

**DOI:** 10.1186/s13104-020-04959-w

**Published:** 2020-02-21

**Authors:** Pedro Pallangyo, Jalack Millinga, Smita Bhalia, Zabella Mkojera, Nsajigwa Misidai, Happiness J. Swai, Naairah R. Hemed, Alice Kaijage, Mohamed Janabi

**Affiliations:** 1 Unit of Research, Jakaya Kikwete Cardiac Institute, P.O Box 65141, Dar es Salaam, Tanzania; 2 Department of Adult Cardiology, Jakaya Kikwete Cardiac Institute, P.O Box 65141, Dar es Salaam, Tanzania; 3 Department of Nursing, Jakaya Kikwete Cardiac Institute, P.O Box 65141, Dar es Salaam, Tanzania

**Keywords:** Heart failure, Nonadherence, Poor adherence, Low adherence, Drug adherence, Medication adherence, Medication compliance, Noncompliance, Tanzania

## Abstract

**Objective:**

Management of heart failure is complex and multifaceted but adherence to medications remains the cornerstone of preventing avoidable readmissions, premature deaths, and unnecessary healthcare expenses. Despite of evidence-based efficacy on anti-failure drugs, poor adherence is pervasive and remains a significant barrier to improving clinical outcomes in heart failure population.

**Results:**

We enrolled 459 patients with diagnosis of heart failure admitted at a tertiary cardiovascular hospital in Dar es Salaam, Tanzania. The mean age was 46.4 years, there was a female predominance (56.5%), 67.5% resided in urban areas and 74.2% had primary education. Of the 419 participants eligible for assessment of medication adherence, 313 (74.7%) had poor adherence and 106 (25.3%) had good adherence. Possession of a health insurance was found to be the strongest associated factor for adherence (adjusted OR 8.7, 95% CI 4.7–16.0, p < 0.001). Participants with poor adherence displayed a 70% increased risk for rehospitalization compared to their counterparts with good adherence (adjusted RR 1.7, 95% CI 1.2–2.9, p = 0.04). Poor adherence was found to be the strongest predictor of early mortality (HR 2.5, 95% CI 1.3–4.6, p < 0.01). In conclusion, Poor medication adherence in patients with heart failure is associated with increased readmissions and mortality.

## Introduction

Cardiovascular disorders (CVD) are responsible for about one-third of all global mortality with over three-quarters of deaths transpiring in the developing world [1]. In spite of the remarkable advances in novel screening techniques and therapeutic directions, the prognosis of heart failure (HF) remains strikingly poor around the globe particularly in the developing nations [2–7]. Owing to its chronic nature, clinical management of HF necessitate long-term use of several drugs to reduce morbidity [8–10] and mortality [11–13]. However, universally low prescription rates of such drugs among patients who require them is observed [14].

Despite of all developments in HF management, adherence plays a pivotal role in attaining maximal therapeutic benefits. Nevertheless, regardless of the assessment tool used or population studied, adherence rates are consistently suboptimal across studies making it a significant public health issue [15–25]. Poor adherence to prescribed regimens is pervasive and results in preventable hospitalizations, premature deaths and unnecessary health care expenditure regardless of the underlying cardiovascular etiology [15–26]. There is dearth of information regarding medication adherence among heart failure population in Tanzania and Sub-Saharan Africa at large. In this prospective cohort study, we sought to explore the adherence pattern, associated factors and outcomes among hospitalized heart failure patients in a tertiary hospital in Tanzania.

## Main text

### Methods

#### Recruitment process and definition of terms

All patients who were hospitalized at Jakaya Kikwete Cardiac Insitute (a tertiary care public teaching hospital) between March and October 2018 with established diagnosis of heart failure (for at least 3 months’ prior enrollment) were consecutively enrolled for this study. Sociodemographic, clinical, laboratory, echocardiographic, and adherence data were gathered using a structured questionnaire during the hospital admission of enrollment. Framingham criteria was used to screen participants for heart failure symptoms and a 2-dimensional echocardiography was utilized for diagnosis reconfirmation. Renal functions were estimated using the Modification of Diet in Renal Disease equation and estimated glomerular filtration rate (eGFR) value of < 60 mL/min/1.73 m^2^ was used to define renal dysfunction. Diagnosis of anemia utilized the WHO criteria i.e. Hemoglobin (Hb) concentration of < 13.0 g/dL and < 12.0 g/dL for males and females respectively. Diabetes was defined by fasting blood glucose levels ≥ 7.0 mmol/L or use of glucose lowering agents. Hypertension was defined as systolic blood pressure (SBP) > 140 mmHg and/or diastolic blood pressure (DBP) > 90 mmHg or use of antihypertensive medications. Total cholesterol level greater than 6.2 mmol/L was used to define dyslipidemia. Hyponatremia, hypokalemia, hypocalcemia, and hypomagnesemia were defined by concentrations < 135 mmol/L, < 3.5 mmol/L, < 2.1 mmol/L and < 0.7 mmol/L respectively. Potassium levels > 5.0 mmol/L was used to denote hyperkalemia. We assessed adherence based on the last time a participant last took her heart failure medications. For the purpose of this study, we defined good adherence as intake of all prescribed heart failure medications within 72 h before the admission of recruitment.

#### Follow-up and study outcomes

Follow-up was conducted through scheduled weekly phone calls and continued through April 2019 with a predetermined stopping point providing a maximum of 180 days of follow-up for each patient after enrollment. Data was censored after completion of follow-up or death, whichever occurred first. A participant was deemed lost to follow-up when despite all attempts couldn’t be reached through phone numbers provided. Our primary outcome measures were rehospitalization and all-cause mortality. We defined rehospitalization as any cardiovascular-related hospital admission following a successful discharge from the hospitalization of enrollment. Early mortality was defined as death during the hospitalization of enrollment.

#### Statistical analysis

All statistical analyses utilized STATA v11.0 software. Pearson Chi square and Student’s T-test were used to compare categorical and continuous variables respectively. Logistic regression analyses was used to assess for factors associated with adherence and predictors of rehospitalization. Factors included in our logistic regression model included age, sex, education level, marital status, employment status, residence, comorbidities and possession of health insurance. Based on their adherence status, participants were compared with respect to survival using Cox proportional-hazards regression model. Differences in survival between the low- and high-adherence groups were compared using the log-rank test. We report Odds ratio (OR), Relative risk (RR) and Hazard ratio (HR) with 95% confidence intervals (CI) and p-values where appropriate. All tests were 2-sided and p < 0.05 was used to denote statistical significance.

### Results

#### Study population

A total of 459 heart failure patients met the inclusion criteria and were enrolled into this study. During follow-up, 40 (8.7%) participants exited; 5 due to incomplete key data and 35 were lost to follow-up. Table 1 displays the baseline characteristics of participants. The mean age of our heart failure cohort was 46.4 ± 18.9 years, there was female preponderance (56.6%) and over two-thirds of all participants resided in urban areas. The mean BMI was 25.1 ± 5.2 and 39.4% of patients were overweight or obese. About 7.2% of participants were in NYHA functional class II while classes III and IV constituted 36.5% and 56.3% respectively. Heart failure with reduced ejection fraction (HFrEF) was present in 284 (67.8%) of participants while 135 (32.2%) had preserved systolic functions (HFpEF). Over a half (52.7%) of participants had a history of hypertension, 13.6% had diabetes, 6.7% were infected with HIV, 51.3% had renal insufficiency and 72.1% were anemic. Echocardiography revealed hypertensive heart disease was the predominant cause of HF (40.1%) followed by dilated cardiomyopathy (27.0%) and rheumatic heart disease (23.2%).

**Table 1:** Baseline characteristics of participants (N = 419)

Characteristic	All	Poor adherence	Good adherence	p-value
	(N = 419)	(n = 313)	(n = 106)	
Age	46.4 (18.9)	45.5 (19.0)	49.1 (18.6)	0.09
Age groups				
< 30	103 (24.6%)	82 (26.2%)	21 (19.8%)	0.19
30–50	129 (30.8%)	96 (30.7%)	33 (31.1%)	0.94
> 50	187 (44.6%)	135 (43.1%)	52 (49.1%)	0.28
Sex				
Male	182 (43.4%)	139 (44.4%)	43 (40.6%)	0.5
Female	237 (56.6%)	174 (55.6%)	63 (59.4%)	
Residence				
Urban	283 (67.5%)	197 (62.9%)	86 (81.1%)	*0.001*
Rural	136 (32.5%)	116 (37.1%)	20 (18.9%)	
Marital status				
Single	100 (23.9%)	82 (26.2%)	18 (17.0%)	0.05
Married	296 (70.6%)	213 (68.1%)	83 (78.3%)	0.05
Divorced/widowed	23 (05.5%)	18 (05.7%)	5 (04.7%)	0.67
Education				
None	16 (03.8%)	12 (03.9%)	4 (03.8%)	0.96
Primary	295 (70.4%)	248 (79.2%)	47 (44.3%)	*<0.001*
Secondary	68 (16.2%)	36 (11.5%)	32 (30.2%)	*<0.001*
University	40 (09.6%)	17 (05.4%)	23 (21.7%)	*<0.001*
Occupation				
None	76 (18.1%)	47 (15.0%)	29 (27.3%)	*<0.01*
Employed/self-employed	311 (74.3%)	250 (79.9%)	61 (57.6%)	*<0.001*
Retired	32 (07.6%)	16 (05.1%)	16 (15.1%)	*0.001*
Body mass index	25.1 (05.2)	24.8 (04.2)	26.0 (07.4)	*0.04*
BMI categories				
Underweight	11 (02.6%)	7 (02.2%)	4 (03.8%)	0.37
Normal	243 (58. 0%)	188 (60.1%)	55 (51.9%)	0.14
Overweight	105 (25.1%)	79 (25.2%)	26 (24.5%)	0.89
Obese	60 (14.3%)	39 (12.5%)	21 (19.8%)	0.06
Health insured				
Yes	93 (22.2%)	32 (10.2%)	61 (57.6%)	*<0.001*
No	326 (77.8%)	281 (89.8%)	45 (42.4%)	
HF etiology				
DCM	113 (27.0%)	78 (24.9%)	34 (32.1%)	0.15
HHD	168 (40.1%)	134 (42.8%)	35 (33.0%)	0.08
RHD	97 (23.2%)	72 (23.0%)	25 (23.6%)	0.9
Others	41 (09.8%)	29 (09.3%)	12 (11.3%)	0.55
Comorbidities				
Hypertension	221 (52.7%)	171 (54.6%)	50 (47.2%)	0.19
Diabetes	57 (13.6%)	39 (12.5%)	18 (17.0%)	0.24
HIV/AIDS	28 (06.7%)	15 (04.8%)	13 (12.3%)	*0.01*
Renal insufficiency	215 (51.3%)	163 (52.1%)	52 (49.1%)	0.59
eGFR < 15	100 (23.9%)	80 (25.6%)	20 (18.9%)	0.16
Anemia	302 (72.1%)	234 (74.8%)	68 (64.2%)	*0.04*
Hb < 8 g/dL	99 (23.6%)	75 (24.0%)	24 (22.6%)	0.77
NYHA class				
II	30 (07.2%)	19 (06.0%)	11 (10.4%)	0.13
III	153 (36.5%)	112 (35.8%)	41 (38.7%)	0.59
IV	236 (56.3%)	182 (58.2%)	54 (50.9%)	0.19
Systolic functions				
Preserved (HFpEF)	135 (32.2%)	96 (71.1%)	39 (28.9%)	0.24
Reduced (HFrEF)	284 (67.8%)	217 (76.4%)	67 (23.6%)	
Admission days	14.0 (13.3)	13.8 (13.4)	14.3 (12.8)	0.74
HF-related hospitalization				
1st	211 (50.4%)	167 (53.3%)	44 (41.5%)	*0.04*
> 1	208 (49.6%)	146 (46.7%)	62 (58.5%)	

#### Medication adherence

Overall, 337 (73.4%) were on angiotensin converting enzyme inhibitors (ACEI), 122 (26.6%) on angiotensin receptor blockers (ARB), 386 (84.1%) on beta-blockers, 432 (94.1%) on diuretics, 395 (86.1%) on aldosterone antagonists, 166 (36.2%) on inotropes and 36 (7.8%) were on digoxin. Of the 419 participants eligible for assessment of medication adherence, 313 (74.7%) had poor adherence and 106 (25.3%) had good adherence. The mean number of days’ participants last took medications before the index hospitalization was 17.7 (± 6.9) days. Among participants with poor adherence, 254 (81.2%) had not taken any of their anti-failure medications within the past 1-week prior admission. Inability to afford medications was the most (87.3%) reported reason for nonadherence. Other reported factors affecting adherence in this cohort included; medication side effects (8.1%), forgetfulness (53.9%), negligence (26.0%), local unavailability of drugs (18.9%) and pill burden (34.4%). Differences in age, sex, marital status, and BMI displayed similar medication adherence patterns, Table 1. However, during bivariate analyses four characteristics including education level, residence, employment status, and health insurance possession showed significant associations with adherence, Table 2. Significant variables then underwent multivariate logistic regression analysis where possession of a health insurance was found to be the strongest associated factor for adherence (OR 8.7, 95% CI 4.7–16.0, p < 0.001), Table 2.

**Table 2:** Factors associated with adherence

Control group	Comparative group	OR	95% CI	p-value	Adj. OR	Adj. 95% CI	Adj. p-value
Age < 50	Age ≥ 50	0.8	0.5–1.2	0.3	–	–	–
Female	Male	1.2	0.7–1.8	0.5	–	–	–
≥ Secondary education	≤ Primary education	5.3	3.3–8.6	*<0.001*	1.9	0.9–4.0	0.07
Married	Single	1.7	1.0–2.8	0.05	–	–	–
Employed	No employment	0.3	0.2–0.5	*<0.001*	1.2	0.6–2.4	0.6
Urban	Rural	2.5	1.5–4.3	*0.001*	2.0	1.1–3.7	*0.03*
No comorbidity	≥ 1 comorbidity	0.9	0.5–1.4	0.56	–	–	–
Health insurance	Not insured	11.9	7.0–20.2	*<0.001*	8.7	4.7–16.0	*<0.001*
HFpEF	HFrEF	1.3	0.8–2.1	*0.28*	–	–	–

#### Rehospitalization and mortality

Overall, 208 (49.6%) patients had a history of a prior cardiovascular-related hospitalization. Despite of similar rehospitalization rates between poor and good-adherence participants at 30-days (35.4% vs 27.2%, p = 0.12) and 90-days (51.8% vs 40.2%, p = 0.07), patients with poor adherence had significantly higher rates of rehospitalization at 180 days (57.5% vs 43.5%, p = 0.03). Overall, participants with poor adherence displayed a 70% increased risk for rehospitalization compared to their counterparts with good adherence (RR 1.7, 95% CI 1.2–2.9, p = 0.04).

**Fig. 1:**
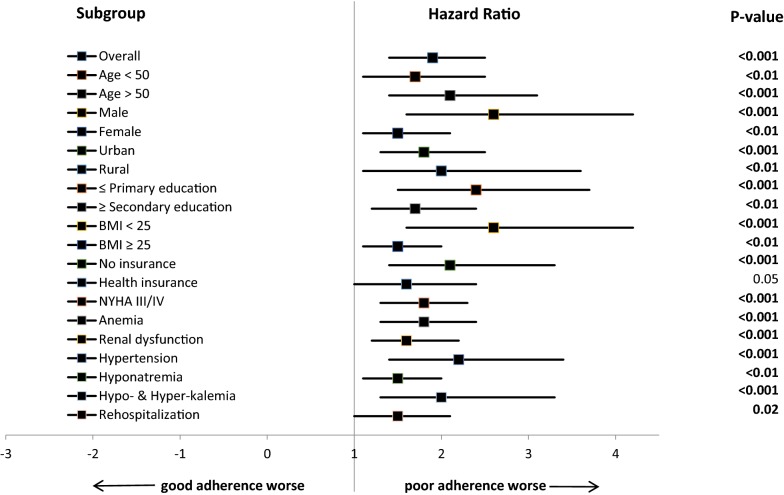
Hazard Ratios for All-cause Mortality by Adherence status. This forest plot shows the hazard ratios (black squares), 95% CIs (horizontal lines), and p-values for the interaction between the All-cause mortality and any subgroup variable by Adherence status

177 (42.2%) patients survived the 180-days of follow-up. The mean survival days was 103.3 ± 74.8 days and participants with good adherence (140.5 ± 63.1 days) displayed a longer survival compared to their poor adherence (90.8 ± 74.3 days) counterparts, p < 0.001. Regardless of the assessment time, participants with poor adherence displayed superior mortality compared to those with good adherence i.e. 37.1% vs 12.3%, 56.6% vs 25.5%, and 65.5% vs 34.9% at 30, 90, and 180 days respectively; all p < 0.001). Additionally, we performed subgroup analyses to assess for all-cause mortality by adherence status. In all 19 characteristics involved in subgroup analyses, participants with poor adherence had inferior survival rates compared to their counterparts with good adherence, Fig. 1. More interestingly, even within the subgroup of those who possessed a health insurance, it was observed that poor adherence participants fared worse compared to good adherence controls, (HR 1.6, 95% CI 1.0–2.4, p = 0.05).

### Discussion

Management of heart failure is complex and multifaceted but adherence to medications remains a fundamental measure to prevent acute exacerbations [27, 28]. Despite of unwavering evidence on the efficacy of anti-failure drugs, poor adherence is common and remains a significant barrier to improving clinical outcomes in heart failure population. Estimates of nonadherence in heart failure patients have varied widely (22–90%) [18, 25, 29–36] in the literature. In this present study, less than one-fifth of participants were categorized as having high adherence. Our rate of nonadherence is skewed to the extreme undesired end of the reported range in the literature.

With regards to reasons for poor adherence, numerous factors have predominated in various studies. For instance, in studies by Toh et al. (71%) and Mujtaba et al. (72.7%), poor medication instructions was the most reported factor [25, 36]. On the other hand, studies by Aggarwal et al. and Dickson et al. found forgetfulness and comorbidities respectively as the leading factors for nonadherence [29, 32]. In this present study, nearly 90% of nonadherent participants reported medication cost as the major barrier to their adherence. These findings are in unison with Dunlay et al. study as far as cost being the most reported factor is concerned, however it was a barrier in a significantly lesser proportion (22%) compared to what we observed [37]. While majority of known risk factors for nonadherence are potentially modifiable, inability to comply due to poverty is not. Owing to this, improving medication adherence in impoverished societies continues to be a very difficult undertaking. It should not be forgotten that such poor societies and their already overwhelmed health sectors continue to struggle with prevention and management of the ever present infectious diseases.

Several studies have demonstrated the repercussions of poor adherence on prognosis of heart failure [16, 32, 33, 38, 39]. Moreover, numerous studies have established the prognostic benefits of interventions to improve adherence [24, 40–50]. In this present study, nearly 60% of participants with poor adherence were rehospitalized within 6-months of enrollment. Our findings are in consonance with several other prospective studies which have produced rehospitalization rates ranging between 20 and 69% [16, 32, 33, 38, 39]. Additionally, intervention studies have uniformly shown that improved adherence is associated with reduction (3–96%) in readmission risk [40, 41, 43, 44, 46–49]. Furthermore, systematic reviews and meta-analyses by Ruppar et al. and Unverzagt et al. revealed a 21% and 10% decreased odds of rehospitalization respectively in the adherence intervention arm [24, 50].

Survival prospects among heart failure patients remain poor all over the globe. Overall, less than half of patients in this study survived the 6-months of follow-up. Nonadherent participants displayed about three times mortality hazard compared to their adherent counterparts. Similar to our findings, intervention studies have shown mortality reduction (2–84%) in favor of adherent participants [40–42, 44–46, 48]. Moreover, two meta-analyses that included over 50 studies each showed a 2% and 11% mortality reduction in favor of the adherence intervention arm [24, 50]. Poor adherence was found to be the strongest predictor of early mortality in this study. To solidify on the significance of adherence in heart failure prognostication, participants with low adherence displayed significantly higher rates of primary outcomes compared to their high adherence counterparts in all subgroup analyses we conducted.

### Conclusions

In conclusion, findings of this present study provide important insight pertaining to medication adherence and its potential in dictating the prognosis of heart failure patients residing in resource-limited settings. Poor adherence in patients with heart failure contributes to a considerable burden on the healthcare system above all increased rehospitalizations and mortality. These findings call for deliberate efforts to ensure that measures to assess and improve adherence are incorporated and become an integral component in routine clinical practice. Furthermore, strategies to improve health insurance acquisition including endeavours to make it a right rather than a privilege is fundamental in improving adherence especially among persons living in impoverished societies.

## Limitations

Medication adherence was ascertained by self-report and thus reporting bias and recall bias could have in some way affected our findings. Prospective comparison of patients receiving adherence intervention versus control would allow a more rigorous evaluation of adherence potential in prognosticating heart failure and should be considered in the future studies in this setting.

## Data Availability

The final version of data set supporting the findings of this paper is submitted together with this manuscript to the editorial committee. All the raw data is included in this manuscript. There are no ethics restrictions preventing the sharing of the raw data.

## Abbreviations

ACE: Angiotensin converting enzyme
ARB: Angiotensin receptor blocker
BMI: Body mass index
CI: Confidence interval
CVD: Cardiovascular disorders
DBP: Diastolic blood pressure
eGFR: Estimated glomerular filtration rate
FBG: Fasting blood glucose
Hb: Hemoglobin
HF: Heart failure
HFpEF: Heart failure with preserved ejection fraction
HFrEF: Heart failure with reduced ejection fraction
HR: Hazard ratio
MDRD: Modification of diet in renal disease
MMAS-8: 8-Item Morisky Medication Adherence Scale
NYHA: New York Heart Association
OR: Odd ratio
RR: Relative risk
SBP: Systolic blood pressure
WHO: World Health Organization
